# The interplay of microRNAs and transcription factors in autophagy regulation in nonalcoholic fatty liver disease

**DOI:** 10.1038/s12276-021-00611-0

**Published:** 2021-04-20

**Authors:** Yumi Kim, Da-Hye Lee, So-Hyun Park, Tae-Il Jeon, Chang Hwa Jung

**Affiliations:** 1 Research Division of Food Functionality, Korea Food Research Institute, Wanju-gun, Jeollabuk-do 55365 Republic of Korea; 2 Department of Biochemistry, Molecular Biology and Biophysics, University of Minnesota, Minneapolis, MN 55455 USA; 3 Department of Food Biotechnology, Korea University of Science and Technology, Daejeon, Republic of Korea; 4 Department of Animal Science, Chonnam National University, Gwangju, Republic of Korea

**Keywords:** Transcription, Non-coding RNAs

## Abstract

The autophagy-lysosomal degradation system has an important role in maintaining liver homeostasis by removing unnecessary intracellular components. Impaired autophagy has been linked to nonalcoholic fatty liver disease (NAFLD), which includes hepatitis, steatosis, fibrosis, and cirrhosis. Thus, gaining an understanding of the mechanisms that regulate autophagy and how autophagy contributes to the development and progression of NAFLD has become the focus of recent studies. Autophagy regulation has been thought to be primarily regulated by cytoplasmic processes; however, recent studies have shown that microRNAs (miRNAs) and transcription factors (TFs) also act as key regulators of autophagy by targeting autophagy-related genes. In this review, we summarize the miRNAs and TFs that regulate the autophagy pathway in NAFLD. We further focus on the transcriptional and posttranscriptional regulation of autophagy and discuss the complex regulatory networks involving these regulators in autophagy. Finally, we highlight the potential of targeting miRNAs and TFs involved in the regulation of autophagy for the treatment of NAFLD.

## Fatty liver disease: the interplay of RNAs and regulatory proteins

Small molecules of ribonucleic acid called microRNAs (miRNAs) and gene-regulating proteins called transcription factors (TFs) control the process of autophagy, which removes unnecessary components of cells and is impaired in nonalcoholic fatty liver disease (NAFLD). This condition is common in people who are obese or overweight. Autophagy is important for maintaining healthy cells and tissues, and is disrupted in several diseases. Researchers led by Chang Hwa Jung at Korea Food Research Institute in Wanju-gun, South Korea, review the interplay of miRNAs and TFs in regulating the activity of genes involved in autophagy and specifically its impairment in NAFLD. Complex regulatory networks being identified offer new insights into autophagy. The authors discuss the potential for developing new therapies for NAFLD using drugs that will interact with the miRNAs and TFs that control autophagy.

## Introduction

Autophagy is important for maintaining intracellular protein homeostasis and for organelle quality control. In the liver, autophagy helps maintain metabolic homeostasis and, consequently, lipid balance, insulin sensitivity, and hepatocyte resistance to injuries, such as oxidative stress and inflammation^1–5^. Accumulating evidence indicates that autophagy is highly relevant to the pathogenesis of nonalcoholic fatty liver disease (NAFLD), including hepatitis and fibrosis, suggesting that modulating autophagy is a potential strategy for the treatment of NAFLD. However, the exact pathophysiological role of and the regulatory mechanisms underlying autophagy in NAFLD remain to be elucidated.

The regulatory network of transcriptional and posttranscriptional factors has recently attracted attention because of its roles in biological processes and in the development of various diseases^6–8^. Autophagy was initially considered a pathway exclusively regulated by cytoplasmic processes; however, over the past decade, a number of microRNAs (miRNAs) and transcription factors (TFs) have been found to control autophagy through the regulation of autophagy-related genes^9,10^. Several miRNAs and TFs that regulate autophagy pathways have been reported to play roles in NAFLD, but the interplay between miRNAs and TFs for the regulation of autophagy is not fully understood. Here, we review the interplay between miRNAs and TFs in regulating autophagy and suggest potential target networks for treating NAFLD.

## The autophagy process

**Fig. 1:**
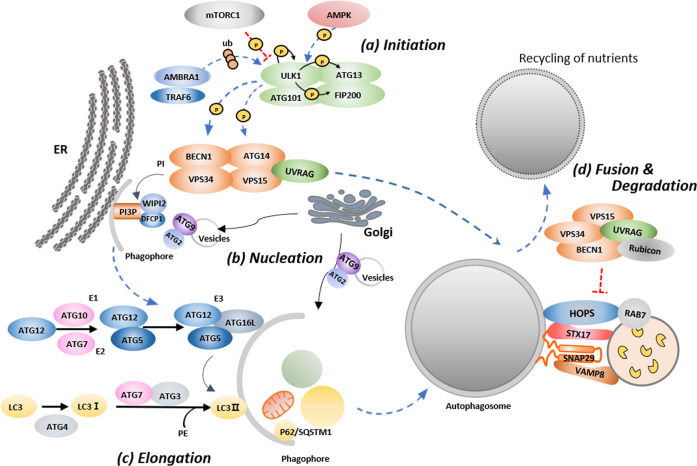
Overview of the autophagy pathway. **a** When autophagy is induced by various stress factors, the Unc-51-like autophagy activating kinase (ULK) complex is activated and initiates autophagy. **b** Activated ULK triggers phosphatidylinositol 3-kinase complex III (VPS34) to form the phagophore at omegasomes. **c** The phagophore is elongated by two ubiquitin-like conjugation systems to form an autophagosome. The first system involves conjugation of autophagy-related protein (ATG)12 to ATG5 in a reaction that requires the E1-like enzyme ATG7 and E2-like enzyme ATG10. The second system involves the conjugation of C-type lectin 3 (LC3) to phosphatidylethanolamine (PE). **d** The mature autophagosome or amphisome binds a lysosome, and the autophagic cargo is degraded by acidic hydrolases in the lysosome and is recycled in the cell as nutrients.

As shown in Fig. 1, the induction of autophagy begins with the Unc-51-like autophagy activating kinase 1 (ULK1) complex formed by the interaction of ULK1 with focal adhesion kinase family-interacting protein of 200 kDa (FIP200), autophagy-related protein (ATG)13, and ATG101^11–13^. Under starved conditions, ULK1 is autophosphorylated and activated; then, ULK1 phosphorylates ATG13 and FIP200 to induce autophagy^14,15^. In contrast, under nutrient-rich conditions, the activated mechanistic target of rapamycin (mTOR) inactivates ULK1 and ATG13 through phosphorylation^16^. AMP-activated protein kinase (AMPK) has also been reported to activate autophagy through the inhibition of mTORC1 and to directly phosphorylate several ATG proteins, including ULK1, Beclin1 (BECN1), and phosphatidylinositol 3-kinase complex III (VPS34)^17–19^. In the early stages of autophagy, activating molecule in Beclin1-regulated autophagy protein 1 (AMBRA1), an autophagy regulatory protein, interacts with the E3 ubiquitinase tumor necrosis factor receptor-associated factor 6 and mediates K63-linked polyubiquitination of ULK1, which enhances ULK1 kinase activity^20,21^.

Phagophores (i.e., isolation membranes) are nucleated at the endoplasmic reticulum (ER)–mitochondria or the ER–plasma membrane contact site. The ATG14L complex is a key component for the nucleation stage. The ATG14L complex consists of VPS34, VPS15, and BECN1, and it is regulated by the kinase activities of ULK1 and 5′ adenosine monophosphate-activated protein kinase (AMPK)^19,22–25^. Activated VPS34 generates phosphatidylinositol 3-phosphate and initiates the formation of the preautophagosomal phagophore^26–28^. Another key protein, involved in the elongation of the phagophore, is ATG9A/B, which is the only known autophagy-related transmembrane protein that circulates between the *trans*-Golgi network and the late endosome through vesicular trafficking^29^. Upon autophagy induction, a portion of the ATG9A pool transiently localizes to autophagic membranes, and its intracellular cycling is positively regulated by both ULK1 and VPS34^30–32^.

Two ubiquitin-like conjugation systems, ATG12 and C-type lectin 3/gamma-aminobutyric acid receptor-associated protein (LC3/GABARAP; ATG8 in yeast), are involved in phagophore expansion^33^. In the first system, ATG12 is covalently conjugated to ATG5, depending on the E1-like enzyme ATG7 and the E2-like enzyme ATG10. Then, the resulting ATG12-ATG5 compound conjugates with ATG16L1 to form the ATG12-ATG5-ATG16L1 complex, which localizes to the outer membrane of the forming autophagosome^34,35^. The second system involves the conjugation of the LC3/GABARAP family proteins to a lipid molecule. LC3/GABARAP is processed at the C-terminal glycine by the cysteine protease ATG4. Then, LC3/GABARAP is activated by ATG7 and is conjugated by ATG3 to the amino group of phosphatidylethanolamine^36–38^. Finally, the ATG16L1-ATG5-ATG12 complex functions as a scaffold for LC3/GABARAP lipidation^39^.

One of the most important molecules in the maturation of the autophagosome and in the fusion with lysosomes is the small GTPase protein RAB7^40^. Fusion with lysosomes is facilitated by membrane tethering factors such as the homotypic fusion and protein sorting (HOPS) complex and soluble N-ethylmaleimide-sensitive factor-activating protein (SNAP) receptor (SNARE) proteins. The HOPS complex interacts with syntaxin17 (STX17), which interacts with SNAP29 and vesicle-associated membrane protein 8 (VAMP8) to promote autophagosome–lysosome fusion^41,42^. UV resistance-associated gene (UVRAG) also promotes the fusion of autophagosomes and lysosomes by binding to VPS16, which is a subunit of the HOPS complex^23,43^. ATG14 also directly binds to STX17-SNAP29 to form a complex with VAMP8 on lysosomes, which further promotes autophagosome–lysosome fusion^44^.

## Roles of autophagy in NAFLD

NAFLD is caused by excessive hepatic fat accumulation that is not caused by significant alcohol consumption^45^. NAFLD is an umbrella term for diseases that includes simple hepatic steatosis, nonalcoholic steatohepatitis (NASH), and cirrhosis. Other than weight loss, there is currently no effective therapy for NAFLD. Studies are currently focused on understanding the molecular mechanisms underlying NAFLD to identify new therapeutic targets, and these studies have revealed a diverse repertoire of potential targets.

In obesity, the disruption of autophagy in the liver and in extrahepatic organs contributes to excessive lipid accumulation in the liver. Singh et al. were the first to suggest that autophagy might be a potential target for the treatment of NAFLD. In their study, *ATG5* knockdown or pharmacological autophagy inhibition significantly increased the cellular triglyceride content^46^. This group suggested that autophagy has essential roles in lipolysis (lipophagy), wherein intracellular lipid droplets are degraded through a self-degradative pathway to alleviate hepatic inflammation and injury. In mouse livers lacking rubicon-like autophagy enhancer (RUBCNL)/Pacer, which promotes autolysosome formation in the late stages of autophagy, autophagic flux is impaired, resulting in lipid accumulation and liver fibrosis^47^. In contrast, enhancing autophagy by overexpressing *Atg7* alleviated hepatic steatosis in ob/ob mice and in high-fat diet (HFD)-fed mice^48^.

**Fig. 2:**
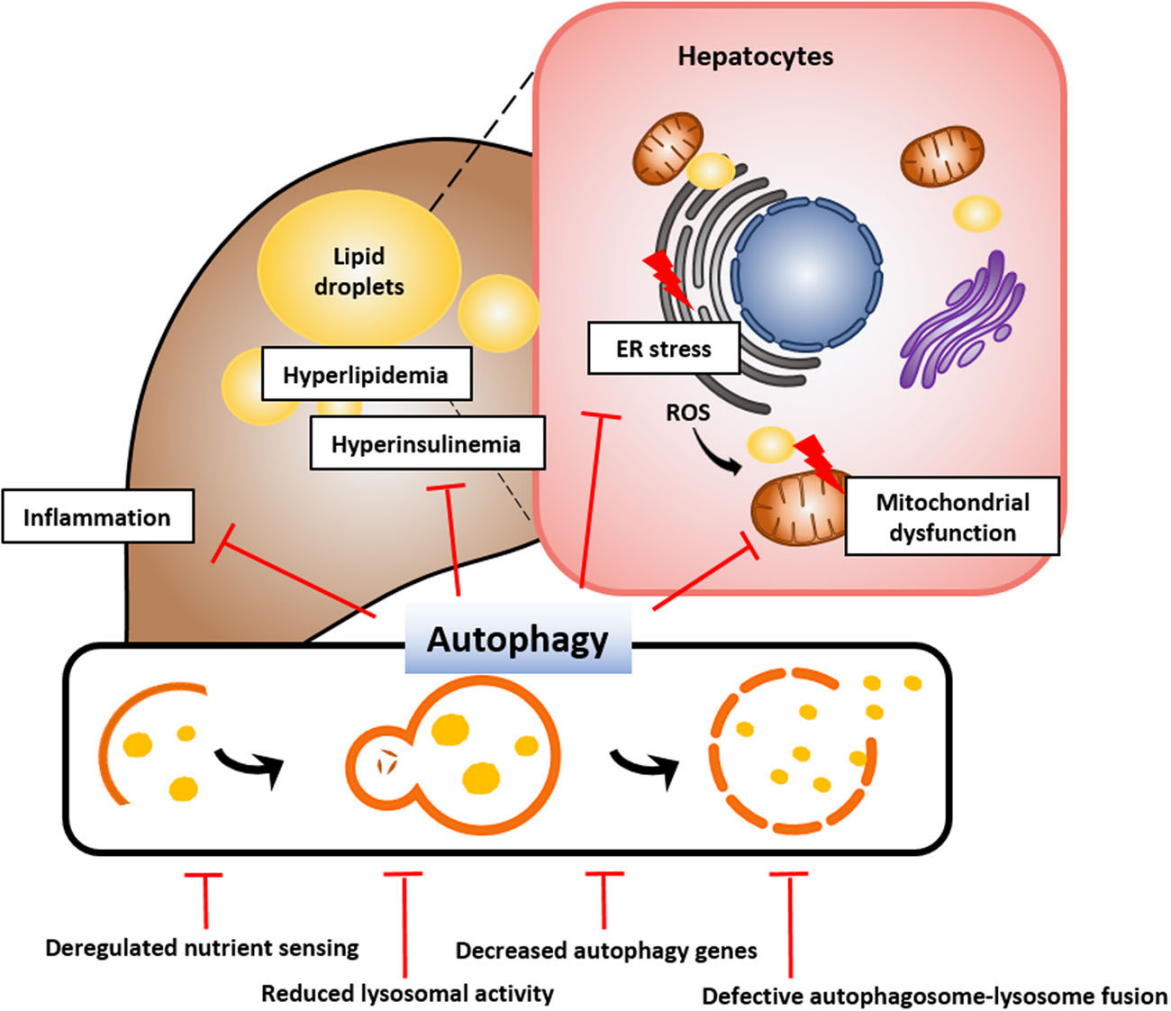
The role of autophagy in nonalcoholic fatty liver disease (NAFLD). Excessive nutrients induce the enlargement of adipose tissue, which increases free fatty acids by promoting lipolysis and inflammatory adipokine secretion. This process leads to lipid accumulation and inflammatory response in the liver, resulting in endoplasmic reticulum (ER) stress and mitochondrial dysfunction. Autophagy, which can inhibit inflammation and promote the degradation of lipid droplets, has the potential as a novel therapeutic target in NAFLD.

Hepatic lipotoxicity is a result of excess accumulation of harmful lipids through the dysregulation of the lipid milieu or intracellular lipid composition. Importantly, lipotoxicity is intimately associated with chronic inflammation and hepatocyte death, which are characteristics of NAFLD/NASH^49^. Lipotoxicity can also contribute to ER stress, mitochondrial dysfunction, and impaired autophagic flux (Fig. 2). For example, lipotoxicity-associated elevation in cytosolic calcium levels interferes with the fusion of autophagosomes and lysosomes, and treatment with calcium channel blockers restores autophagic flux and suppresses obesity-induced accumulation of protein aggregates^50^. Intake of a HFD stimulates ER stress, which can induce an inflammatory response. Suppressed autophagy in the liver can further worsen ER stress and inflammation. These findings indicate that a decrease in hepatic autophagy correlates with hepatic inflammation in NAFLD. In line with this finding, autophagy can protect the liver from lipotoxicity in NAFLD. Sequestosome 1 (SQSTM1 or p62)-mediated ULK1 activation induces autophagy and plays a hepatoprotective role through the degradation of Kelch-like ECH-associated protein 1and the activation of nuclear factor erythroid 2-related factor 2 under saturated fatty acid-induced lipotoxic conditions^51^.

Various studies have revealed some of the molecular mechanisms involved in the regulation of autophagy in NAFLD. Autophagy activity has been seen to decrease as an effect of the reduced expression of autophagy-related genes such as *Atg7*, *Ulk1*, and *Atg9*^48,52^. Impaired autophagic flux caused by a decrease in lysosomal activity has been shown to be critical for the degradation of autophagosomes. Autophagic degradation is impaired owing to reduced lysosomal acidification and lysosomal proteolytic activity, such as those facilitated by hepatic cathepsin B and L^53–55^. Defects in autophagosome–lysosome fusion also cause autophagy disruption in NAFLD. Changes in intracellular lipid content can affect the overall activity of intracellular proteolytic pathways by inhibiting autophagosome–lysosome fusion^56^. Rubicon is a Beclin1-binding negative regulator of autophagosome–lysosome fusion. Rubicon is posttranscriptionally upregulated in HepG2 cells treated with saturated fatty acids and in the liver of HFD-fed mice^54^. In a recent chemokine study, osteopontin, a chemokine-like phosphorylated glycoprotein, promoted NAFLD progression by impairing autophagy^57^. Osteopontin secreted from fatty liver inhibits autophagosome–lysosome fusion by binding the integrins αVβ3 and αVβ5, and autophagic flux is recovered when αVβ3 and αVβ5 are blocked using specific antibodies. Another mechanism of autophagy regulation involves the deregulation of nutrient sensing. A representative example is the regulation of autophagy by mTORC1 through the downregulation of the ULK1-ATG13-FIP200 and VPS34-ATG14-BECN1 complexes. In fatty liver disease, mTORC1 is aberrantly activated and inhibits autophagy; it also contributes to the regulation of de novo lipogenesis through the upregulation of TFs such as sterol regulatory element-binding protein 1^58^. In contrast, AMPK negatively regulates mTORC1 and induces autophagy through ULK1 or BECN1 phosphorylation^59,60^. Most studies show that AMPK activity is reduced in the fatty liver and that the activation of AMPK using genetic or pharmacological approaches reprograms lipid metabolism, thereby reducing hepatic steatosis^61^. The regulation of autophagy through AMPK also contributes to a reduction in hepatic steatosis to some extent.

## MiRNAs that target autophagy-related genes in NAFLD

**Fig. 3:**
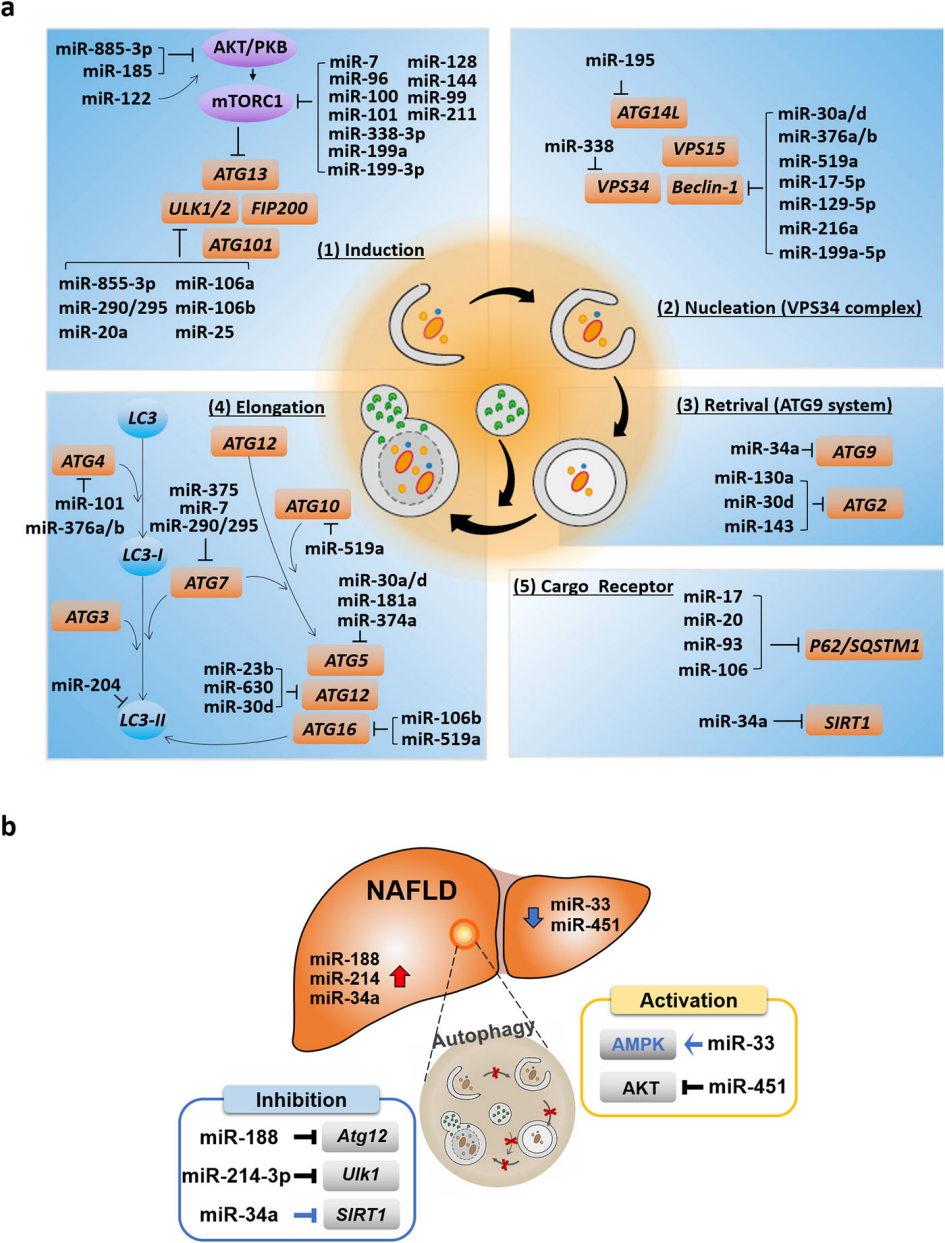
Schematic representation of core autophagy-related proteins and their regulation by miRNAs. **a** Summary of miRNAs that target key proteins in different stages of autophagy. **b** miRNAs regulating autophagy-related genes in NAFLD. Blue indicates that the correlation between the miRNA-targeted protein and autophagy is not clearly demonstrated in NAFLD.

miRNAs are noncoding RNAs consisting of approximately 22 nucleotides^62^. They play roles in the posttranscriptional regulation of gene expression^63^. Approximately 60% of mammalian genes are known to be regulated by miRNAs^64^. Recent studies have reported that miRNAs also play roles in the regulation of autophagy; specifically, they regulate the expression of several key proteins in the various stages of the autophagy pathway. Therefore, we summarize representative miRNAs involved in the regulation of each stage of the autophagy pathway and discuss these mechanisms in the context of NAFLD (Fig. 3a).

In the initial stages of autophagy, the ULK1/2 complex is directly controlled by mTORC1. A number of miRNAs targeting mTORC1 have been reported in various cell and tissue types. miR-7 induces autophagy through the negative regulation of mTORC1^65^. In contrast, miR-199a, miR-338, miR-96, miR-100, miR-101, miR-128, miR-144, miR-99, and miR-211 have been suggested as potential positive regulators of autophagy through their direct inhibition of mTORC1^66–72^. Several miRNAs have been reported to directly target the ULK1/2 complex. miR-885-3p was found to directly target *ULK2*, whereas the activation of miR-106a/b and miR-20a was found to inhibit autophagy through the downregulation of *ULK1* expression^73^. In glucose-deficient melanoma cells, the miR-290/295 cluster code inhibited *ULK1* expression, thereby inhibiting autophagy and endowing tumor cells with a survival advantage^74^. miR-25 in breast cancer cells also targets *ULK1* and inhibits autophagy^75^. miRNAs also regulate autophagosome nucleation stage autophagy genes, such as *VPS34*, *BECN1*, and *ATG14L*. miR-30a/d, miR-376a/b, miR-519a, miR-17-5p, miR-129-5p, miR-199a-5p, and miR-216a inhibit *BECN1* expression^76–81^. miR-338 inhibits the expression of *VPS34*^82^, and miR-195 inhibits *ATG14*-mediated autophagy^83^. Therefore, the suppression of these miRNAs promotes autophagy. *Atg9* is the only transmembrane protein associated with the Golgi complex in the autophagy pathway, and it is targeted by miR-34a in the negative regulation of autophagy^84^. In addition, miR-130a, miR-30d, and miR-143 suppress *ATG2* expression^85–87^.

The ubiquitin-like conjugation system for vesicle expansion is composed of several proteins, such as ATG7, ATG10A, ATG3, ATG12, ATG5, ATG16L1, and LC3. miR-375, miR-17, and miR-290/295 target *ATG7*, resulting in decreased *ATG7* expression, thereby attenuating autophagy^74,88,89^. miR-30a, miR-181a, miR-374a, and miR-30d are direct regulators of *ATG5*^82,85,90,91^. Moreover, miR-30d, miR-23b, and miR-630 are potential regulators of *ATG12*^78,85,92^. miR-106b and 519a target *ATG16L1* expression in intestinal epithelial cells^78,93^. *LC3B* levels and autophagy are diminished by miR-204^94,95^. The miR-101 and miR376 family members miR-376a and miR-376b can negatively control *ATG4* expression^68,81,96^. miR-519a has been shown to modulate E2-like enzyme *ATG10A* levels; however, the role of this interaction on autophagy has not yet been investigated^78^.

Several autophagy receptors have been discovered, including SQSTM1/p62, neighbor of breast cancer type 1 susceptibility protein gene 1 protein (NBR1), calcium-binding and coiled-coil domain-containing protein 2 (NDP52), optineurin (OPTN), and B-cell lymphoma 2/adenovirus E1B 19 kDa protein-interacting protein 3-like (NIX)^97–101^. Because these receptors are delivered to the autophagosome along with cellular cargo, autophagy receptor degradation is commonly used as a marker for autophagic degradation^102^. Members of a family of miRNAs that share a common seed sequence, including miR-17, miR-20, miR-93, and miR-106, were identified as direct regulators of *SQSTM1/p62*^103,104^.

Several studies have shown that changes in miRNA profiles are associated with a number of liver diseases. The expression profiles and signatures of these miRNAs can be used to distinguish between liver diseases with different etiologies and stages, from NAFLD to hepatocellular carcinoma (HCC)^105^. Recently, autophagy-related miRNAs were found to play important roles in NAFLD. We present a summary of the current knowledge on the regulation of autophagy by miRNAs and how miRNAs modify major autophagic effector proteins in NAFLD in Fig. 3b. Several studies have shown that autophagy regulation-related miRNAs, such as miR-214-3p, miR-188, and miR-34a, are expressed at high levels in NAFLD patients or in mouse models^52,106^. In our previous study, we observed that miR-214-3p has an inhibitory role in autophagy and is upregulated in HFD-induced fatty liver^52^. Therefore, an anti-mimic of miR-214-3p alleviated NAFLD by activating autophagy and depended on the upregulation of *Ulk1*. miR-188, known to target and suppress *Atg12*, was reported to be upregulated in the liver of obese mice^107^. According to Liu et al., the negative effects of miR-188 on the lipid and glucose metabolism-mediated autophagy pathway can be reversed by targeting *Atg12*. miR-34a is significantly upregulated in the liver of tissue with NAFLD^106^. miR-34a, a direct inhibitor of nicotinamide adenine dinucleotide-dependent protein deacetylase sirtuin 1 (*SIRT1)*, is a potential biomarker of NAFLD^108^. Although the role of this interaction in the regulation of autophagy has not been observed, SIRT1 can have a direct role in autophagy regulation and affect the expression of autophagy-associated genes, including *MAP1LC3A* (*LC3II*), *ATG5*, and *ATG7*^109,110^. Stacchiotti et al. showed that therapeutic melatonin plays a role in NAFLD through the downregulation of miR-34a-5p and the activation of autophagy; however, they did not investigate the specific autophagy-related genes that were directly regulated by miR-34a^111^.

miRNAs can also directly or indirectly activate autophagy. miR-33 and miR-451 can activate autophagy, and these miRNAs are expressed at low levels in NAFLD^112–114^. The specific role of miR-33 in direct autophagy regulation in NAFLD remains unknown. However, Ghareghani et al. found that miR-33a and miR-33b activated autophagy by enhancing the activation of AMPK in HFD-fed mice^112^. miR-451 levels were reduced in the liver tissue of the obese mice, and miR-451 was found to be a negative regulator of migration inhibitory factor (MIF)^115^. Thus, Tang et al. found that MIF was upregulated in mice fed an HFD because the miR-451 level was reduced. Their results also indicated that MIF was reduced by miR-451 mimic transfection, which induced the inactivation of protein kinase B (Akt) and activation of LC3II. The same results were indicated upon MIF knockout. In contrast, increased Akt and reduced LC3II expression were induced by miR-451 silencing. In addition, Hur et al.^113^ showed that the downregulation of miR-451 in a NAFLD mouse model inhibited the production of fatty acid-induced proinflammatory cytokines through the AMPK/AKT pathway. Important miRNAs that can prevent or alleviate NAFLD can be modulated through autophagy activation.

## TFs that target autophagy-related genes in NAFLD

**Fig. 4:**
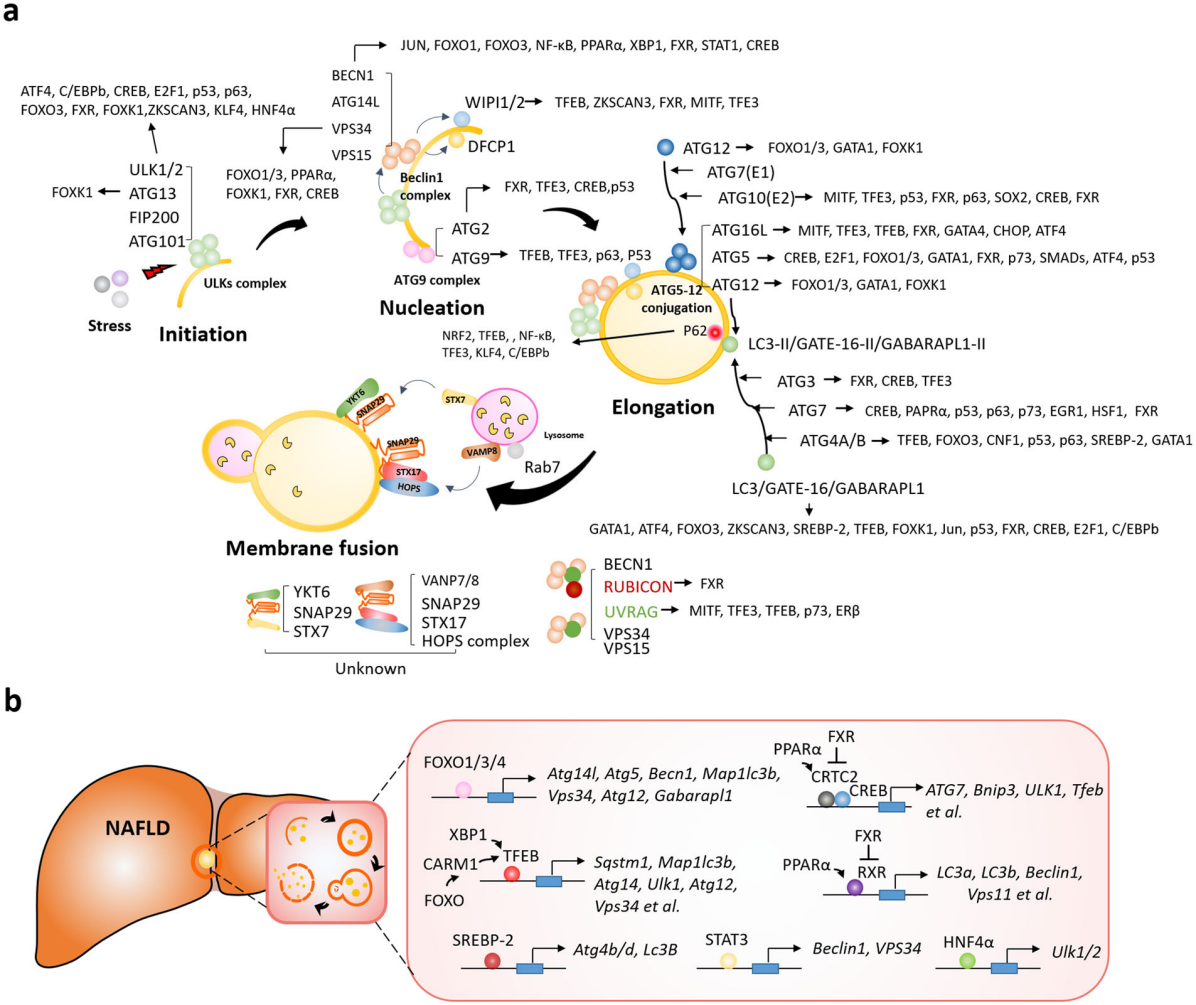
Transcription factors (TFs) that target autophagy-related genes in nonalcoholic fatty liver. **a** Summary of TFs targeting autophagy-related genes in the different stages of autophagy progression. **b** Transcription factors directly involved in regulating autophagy-related genes in NAFLD.

Currently, approximately 32 TFs involved in the regulation of autophagy-related genes have been reported; the most representative TFs are transcription factor EB (TFEB), cAMP response element-binding protein (CREB), forkhead box O proteins (FOXOs), farnesoid X receptor (FXR), and peroxisome proliferator-activated receptor alpha (PPARα) (Fig. 4a). We present a summary of the most representative TFs associated with autophagy-related genes in NAFLD in Fig. 4b.

TFEB acts as a master regulator of lysosomal biogenesis, autophagy, lysosomal exocytosis, lipid catabolism, energy metabolism, and the immune response^116^. TFEB is a member of the microphthalmia/transcription factor E (MIT/TFE) family of TFs, and it recognizes and binds to the E-box sequence (CANNTG)^117^. For efficient DNA binding, TFEB dimerizes with itself or with other MIT/TFE family members, such as IGHM Enhancer 3 (TFE3), transcription factor EC (TFEC), and microphthalmia-associated TF (MITF)^118^. TFEB regulates the expression of key genes in autophagy, such as *BECN1*, *WIPI1* (which encodes WD repeat domain phosphoinositide-interacting protein 1), *GABARAP*, *ATG16 L*, *ATG5*, and *UVRAG*^119^. TFEB activity is regulated by phosphorylation (at S122, S142, and S211) by mTORC1; phosphorylated TFEB is sequestered into the cytoplasm, and the induction of transcription of the target gene is suppressed^120,121^. In contrast, when mTORC1 activity is inhibited during nutrient deficiency, TFEB is dephosphorylated and rapidly migrates to the nucleus, where it binds the promoter of the target gene. Similarly, mTORC1 also inhibits the nuclear localization of TFE3 and MITF, effectively inhibiting the transcriptional induction of autophagy genes^122^. In HFD-fed mice that overexpress TFEB, lipid accumulation in the liver was reduced, and metabolic syndrome was attenuated during a 24-hour starvation period^123^. In line with the role of TFEB in energy control, modest restriction of calorie consumption and regular exercise may be promising approaches for treating early-stage NAFLD, whereas therapeutic agents that target TFEB may be promising for preventing the development of NAFLD into NASH.

FOXO1, which has an important role in both glucose and lipid metabolism, promotes lipid droplet breakdown through transcriptional activation of hepatic autophagy by inducing a number of autophagy-related genes, including *Atg5*, *Atg14*, *Vps34*, *Atg12*, and *Gabarap1*^124,125^. Under nutrient starvation conditions, AMPK phosphorylates FOXO3 in the nucleus, and FOXO3 inhibits the transcription of S-phase kinase-associated protein 2 (*SKP2*), which is an SKP1, Cullins, F-box protein (SCF) E3 ubiquitin ligase complex critical for the degradation of coactivator-associated arginine methyltransferase 1 (CARM1). Stabilized CARM1 promotes histone H3 Arg17 demethylation and binds TFEB as a coactivator, thereby promoting the expression of autophagy-related genes^126^. In addition, spliced X-box binding protein 1 (sXBP1), the key TF that promotes the adaptive unfolded protein response, regulates autophagy-related genes by occupying the −743 to −523 site of the TFEB promoter in the liver under fasting conditions^127^. However, evidence showing that these mechanisms can be targeted for NAFLD therapy is still insufficient.

The fasting transcriptional activator CREB upregulates autophagy genes, including *ATG7* and *ULK1*, by recruiting CREB-regulated transcription coactivator 2 (CRTC2) in hepatocytes. On the other hand, FXR transrepresses autophagy-related genes by disrupting the CREP-CRTC2 complex, suggesting that the FXR-CREB axis is a key physiological switch for regulating autophagy^128^. The CREB–CRTC2 complex upregulates the expression of TFEB, in addition to autophagy-related genes. Similar to the way CRTC2 competes with FXR for binding CREB in proximal autophagy-related genes, PPARα competes with FXR for binding the DR1 response element in the promoters of these genes, and thus, PPARα acts as a ligand-dependent transactivator^129,130^.

SREBP-2, a major transcriptional regulator of cholesterol metabolism, targets lipid metabolic processes, and it also directly activates autophagy-related genes during cellular sterol depletion in the liver^131^. Signal transducer and activator of transcription 3 (STAT3) also regulates autophagy-related genes. Interleukin (IL)-17A inhibits autophagy by activating STAT3 in hepatic fibrosis; in turn, STAT3 downregulates the expression of *BECN1* and *VPS34*, which are involved in the development of hepatic fibrosis^132^. On the other hand, in methionine/choline-deficient diet-induced NASH, autophagy-related proteins such as BECN1 and SQSTM1/p62 are upregulated, but autophagic flux is impaired by hypoxia-inducible factor-1 alpha (HIF-1α), which induces liver steatosis and inflammation^133^. It has recently been reported that hepatocyte nuclear factor 4 alpha (HNF4α), which is essential for hepatocyte differentiation and has an important role in liver function, also has an important role in autophagy. Hnf4α expression was reduced in the fatty liver of mice fed an HFD for a prolonged period and activated autophagy by directly regulating *Ulk1* expression in the liver^52^.

## The interplay of miRNAs and TFs in autophagy regulation in NAFLD

miRNAs and TFs often play coordinating roles in the regulation of various cellular processes via a complex signal transduction network in the liver^134,135^. For example, in human HCC cells, miR-223 and FOXO3a modulate doxorubicin-induced cytoprotective autophagy, contributing to chemoresistance. However, miR-223 overexpression suppresses Foxo3a-modulated autophagy, which enhances doxorubicin sensitivity in a mouse xenograft model of HCC, suggesting that this miRNA/TF axis is an important mechanism for drug resistance development in HCC^136,137^. TFEB-mediated transactivation is also regulated by miR-30-5p, which suppresses TFEB-dependent downstream gene expression by binding to coordinated lysosomal expression and regulation element, leading to the inhibition of lysosomal biogenesis and autophagy in mouse liver^138^.

Accumulating evidence shows that miR-34a is involved in NAFLD, and miR-34a expression is increased in NASH patients and in obese or diabetic mice^108,139,140^. miR-34a promotes hepatic steatosis through the suppression of various TFs, such as HNF4α^141^, PPARα, and SIRT1, which promote the expression of autophagy-related genes^142–144^. These observations suggest that the miR-34a/TF axis may inhibit NAFLD progression through transcriptional regulation of autophagy^52,109,130,145^. Interestingly, miR-34a is directly activated by nuclear receptor liver X receptor-α, a ligand-dependent TFr involved in hepatic cholesterol metabolism. miR-34a also inhibits *Atg4B* and *Rab8b*, which regulate autophagic flux, leading to the progression of hepatic steatosis^146,147^. Considering the role of LXR in cholesterol homeostasis and that increased hepatic free cholesterol is associated with the development of NASH from NAFL in obese mice, cross-talk between TFs, miR-34a, and autophagy may be important for controlling NASH development.

**Fig. 5:**
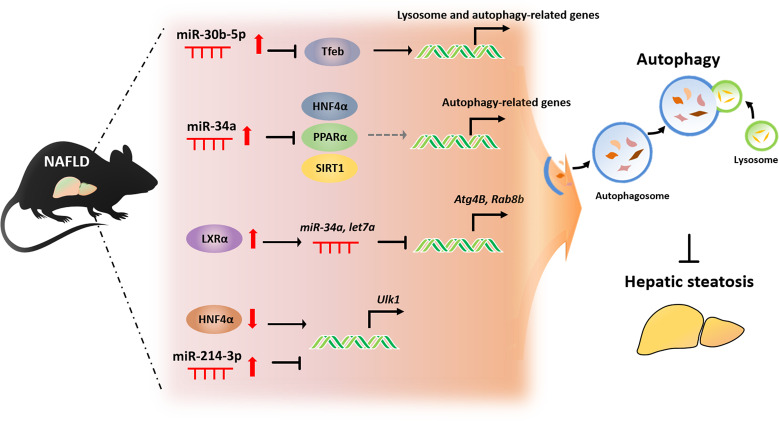
The interplay of miRNAs and transcription factors (TFs) in regulating autophagy in the fatty liver. miR-30b-5p and miR-34a downregulate autophagy-related gene expression by directly inhibiting TFs, such as transcription factor EB (TFEB), Hnf4α, peroxisome proliferator-activated receptor alpha (PPARα), and NAD-dependent protein deacetylase sirtuin 1 (SIRT1). Liver X receptor-α (LXRα) transcriptionally activates *miR-34a* and *let7a*, which directly target the 3’UTR of *Atg4B* and *Rab8b*. miR-214-3p and HNF4α reciprocally regulate *Ulk1* expression.

Recently, we reported certain miRNAs and TFs that regulate autophagy in the development of HFD-induced fatty liver. As shown in Fig. 5, we found that miR-214-3p and HNF4α modulated *Ulk1* expression and autophagy in hepatocytes^52^. Our results indicate that autophagy in the fatty liver was attenuated only when mice were fed a 45% HFD for a prolonged period, which led to a significant reduction in the expression of autophagy-related genes, such as *Ulk1*. This downregulation of autophagy was caused by increased miR-214-3p and decreased HNF4α levels in hepatocytes. miR-214-3p negatively regulates *Ulk1* expression through direct binding of the 3´-UTR sequence of *Ulk1*, and HNF4α induces autophagy by directly binding *Ulk1*, promoting its transcription. Thus, both miR-214-3p and HNF4α act as regulatory factors of *Ulk1* expression. Although the inhibition of miR-214-3p in the fatty liver appears to restore HNF4α expression, miR-214-3p does not directly regulate HNF4α, suggesting that miR-214-3p and HNF4α independently regulate *Ulk1* expression. The interplay between miR-214-3p and HNF4α and their involvement in the regulation of autophagy in the fatty liver are summarized in Fig. 5. Taken together, we propose that miR-214-3p and HNF4α are potential targets for NAFLD therapy.

## Concluding remarks

Studies on the molecular regulation of autophagy mainly focus on how autophagy-related proteins bind and function in the cytoplasm. However, as described in this review, there has been growing interest in understanding the roles of miRNAs and TFs that influence the autophagy pathway. Transcriptional regulation of autophagy may be associated with posttranslational regulation to coordinate the fine-tuning of autophagic flux, especially in cells under chronic stress. In addition, the transcriptional induction of autophagy-related genes may prevent the depletion of the corresponding proteins under stress conditions, as the degradation of autophagy-related proteins is enhanced under normal conditions. Thus, it is important to identify novel co-regulatory networks of miRNAs and TFs that contribute to autophagy, characterize these networks within the context of autophagy-related proteins, and determine how these networks are perturbed in NAFLD. Given the studies that demonstrate the benefits of targeting autophagy for treating NAFLD, transcriptional regulation of autophagy is expected to have similar benefits for the treatment of NAFLD. The effects of the currently available NAFLD drugs on autophagy are not yet known; however, we believe that selective induction of autophagy will be a useful therapeutic strategy for NAFLD.
